# Porphyromonas gingivalis Tyrosine Phosphatase Php1 Promotes Community Development and Pathogenicity

**DOI:** 10.1128/mBio.02004-19

**Published:** 2019-09-24

**Authors:** Young-Jung Jung, Daniel P. Miller, John D. Perpich, Zackary R. Fitzsimonds, Daonan Shen, Jun Ohshima, Richard J. Lamont

**Affiliations:** a Department of Oral Immunology and Infectious Diseases, University of Louisville School of Dentistry, Louisville, Kentucky, USA; KUMC

**Keywords:** microbial communities, periodontitis, tyrosine phosphatase

## Abstract

Protein-tyrosine phosphorylation in bacteria plays a significant role in multiple cellular functions, including those related to community development and virulence. Metal-dependent protein tyrosine phosphatases that belong to the polymerase and histindinol phosphatase (PHP) family are widespread in Gram-positive bacteria. Here, we show that Porphyromonas gingivalis, a Gram-negative periodontal pathogen, expresses a PHP protein, Php1, with divalent metal ion-dependent tyrosine phosphatase activity. Php1 tyrosine phosphatase activity was attenuated by mutation of conserved histidine residues that are important for the coordination of metal ions and by mutation of a conserved arginine residue, a key residue for catalysis in other bacterial PHPs. The *php1* gene is located immediately downstream of the gene encoding the bacterial tyrosine (BY) kinase Ptk1, which was a substrate for Php1 *in vitro*. Php1 rapidly caused the conversion of Ptk1 to a state of low tyrosine phosphorylation in the absence of discernible intermediate phosphoforms. Active Php1 was required for P. gingivalis exopolysaccharide production and for community development with the antecedent oral biofilm constituent Streptococcus gordonii under nutrient-depleted conditions. In contrast, the absence of Php1 had no effect on the ability of P. gingivalis to form monospecies biofilms. *In vitro*, Php1 enzymatic activity was resistant to the effects of the streptococcal secreted metabolites pABA and H_2_O_2_, which inhibited Ltp1, an enzyme in the low-molecular-weight (LMW) phosphotyrosine phosphatase family. Ptk1 reciprocally phosphorylated Php1 on tyrosine residues 159 and 161, which independently impacted phosphatase activity. Loss of Php1 rendered P. gingivalis nonvirulent in an animal model of periodontal disease. Collectively, these results demonstrate that P. gingivalis possesses active PHP and LMW tyrosine phosphatases, a unique configuration in Gram-negatives which may allow P. gingivalis to maintain phosphorylation/dephosphorylation homeostasis in multispecies communities. Moreover, Php1 contributes to the pathogenic potential of the organism.

## Abstract (precis)

Periodontal diseases are among the most common infections of humans and are also associated with systemic inflammatory conditions. Colonization and pathogenicity of P. gingivalis are regulated by signal transduction pathways based on protein tyrosine phosphorylation and dephosphorylation. Here, we identify and characterize a novel component of the tyrosine (de)phosphorylation axis: a polymerase and histindinol phosphatase (PHP) family enzyme. This tyrosine phosphatase, designated Php1, was required for P. gingivalis community development with other oral bacteria, and in the absence of Php1 activity P. gingivalis was unable to cause disease in a mouse model of periodontitis. This work provides significant insights into the protein tyrosine (de)phosphorylation network in P. gingivalis, its adaptation to heterotypic communities, and its contribution to colonization and virulence.

## INTRODUCTION

Periodontal diseases, which involve inflammatory-based destruction of the tissues that surround and support the teeth, are initiated by multispecies communities in the gingival compartment. Periodontal diseases and periodontal organisms are also epidemiologically and mechanistically associated with serious systemic conditions, such as coronary artery disease and Alzheimer’s disease (1–3). In the gingival compartment, Porphyromonas gingivalis resides within multispecies communities, and interactions among community participants define spatial organization and overall metabolic and pathogenic properties. Such interactions are based on coadhesion and small-molecule-based communication, and these processes are extensively studied (4–6). Less is known, however, regarding the signal transduction pathways within bacterial cells that relay information derived from the microenvironment and from community partner species.

While not a numerically dominant member of the periodontal microbiome, the keystone pathogen P. gingivalis can elevate the nososymbiocity (or pathogenic potential) of periodontal communities through interactions with accessory pathogens and pathobionts (7, 8). Streptococcus gordonii is a major partner species of P. gingivalis, and in the murine oral periodontitis model coinfection with both species results in greater alveolar bone loss than infection with either species alone (9, 10). Community development between the two bacterial species is mediated by coadhesion and chemical communication based on streptococcal metabolites such as pABA (11). Our previous studies have shown that virulence and community development of P. gingivalis are controlled by Ptk1, a BY kinase of P. gingivalis (12). Ptk1 is a substrate of Ltp1, a low-molecular-weight protein tyrosine phosphatase (LMW-PTP) (12–14), forming a cognate kinase-phosphatase pair, but their genes are remotely located (*ptk1* [PGN_1524 and PGN_RS07275] and *ltp1* [PGN_0491 and PGN_RS02345]), an uncommon arrangement in Gram-negative bacteria, indicating the possibility of additional phosphatases or kinases encoded by genes adjacent to the kinase or phosphatase (15).

The operon in which *ptk1* is located in P. gingivalis consists of three genes (PGN_1523 [PGN_RS07270], *ptk1*, and PGN_1525 [PGN_RS07280]) (12). Bioinformatically, PGN_1525 has histidinol phosphatase-like motifs belonging to the polymerase and histidinol phosphatase family of protein tyrosine phosphatases (PHP-PTP). Most PHP-PTPs are found in Gram-positive bacteria, such as Streptococcus pneumoniae, Bacillus subtilis, and Lactobacillus rhamnosus, and they are involved in the control of capsular polysaccharide production, DNA metabolism, cell division, and bacterial invasiveness (16–20). Thus far, the only Gram-negative organism that has been demonstrated to have an active tyrosine phosphatase in the PHP family is Myxococcus xanthus, in which PhpA is responsible for negative control of extracellular polysaccharide (EPS) production and spore formation (21). In this study, we demonstrate that PGN_1525 encodes an active PHP family tyrosine phosphatase, designated here Php1, which can act on Ptk1 and participate in the control of community development along with virulence in an animal model of periodontal disease.

## RESULTS

### Comparative sequence analysis and enzymatic characterization of Php1.

**FIG 1:**
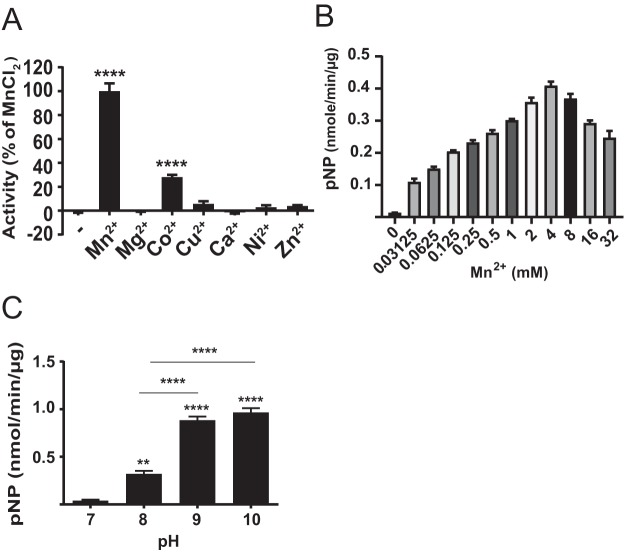
Characterization of Php1 phosphatase activity. (A) Recombinant Php1 was incubated with 50 mM *p*NPP in a reaction mixture (pH 8.0) containing 1 mM (final concentration) the indicated ions. (B) Effect of Mn^2+^ concentration on Php1 activity against *p*NPP. (C) Php1 activity against *p*NPP measured at different pH values of the reaction mixture. Reactions were performed in triplicate, and data are expressed as means ± standard errors (SE). **, *P < *0.01; ****, *P < *0.001.

In P. gingivalis 33277, PGN_1525, a gene immediately downstream of *ptk1*, encodes a predicted 28-kDa protein containing a PHP domain (PF02811). BLASTP analysis revealed that it shares significant homology with PHP-PTP proteins of other bacterial species, including all the invariant histidine, aspartate, and arginine residues in four conserved motifs (see Fig. S2A in the supplemental material). Moreover, homology modeling (Fig. S2B) suggests strong structural conservancy with YwqE, a PHP-PTP of Bacillus subtilis. The tertiary conformation is highly homologous along with conserved residues H27, H64, H155, and R158, which form a catalytic pocket. Thus, here the PGN_1525 protein is designated Php1. Previous studies have shown that divalent metal ions are essential cofactors for PHP tyrosine phosphatase enzyme activity (21–23). The active site of the prototypic PHP-PTP enzymes YwqE and CpsB (from Streptococcus pneumoniae) contains a cluster of three metal ions bound adjacent to each other between β-sheets (22, 24). The phosphatase activity of recombinant Php1 also was dependent on the presence of Mn^2+^ or, to a lesser degree, Co^2+^ (Fig. 1A), as described for CpsB (25), and PhpA from M. xanthus (21). YwqE is also Mn^2+^ dependent but is also stimulated by Cu or Zn ions, and different members of the enzyme family differ in their pattern of sensitivity to cations other than Mn^2+^ (25, 26). Php1 activity was enhanced by increasing concentrations of Mn^2+^ up to 4 mM but was decreased at higher Mn^2+^ concentrations (Fig. 1B). When the effect of pH on the phosphatase activity of Php1 was examined, activity was enhanced by increasing pH within a range of pH 8.0 to 10.0, while activity was not significantly above background levels at pH 7.0 (Fig. 1C). Hence, *in vitro*, Php1 is only active under basic conditions, as described for this family of enzymes (26).

10.1128/mBio.02004-19.2 FIG S2 (A) Amino acid sequence alignment of P. gingivalis Php1 and its homologues from other bacteria. The four conserved motifs of the PHP domain (PF02811) are highlighted with yellow, green, blue, and magenta. Identical residues among PHP proteins are indicated with an asterisk. Php1 (RefSeq accession no. WP_012458344) shares 25, 27, 25, 28, 32, 29, and 37% amino acid identity and 47, 46, 47, 47, 46, 46, and 55% amino acid similarity with Wzb (AAW22448) of *Lactococcus rhamnosus* (23), PtpZ (P96717) of Bacillus subtilis (26), EpsC (NP_053031) of L. lactis (58), EpsB (AAK61896) of Streptococcus thermophilus (59), CpsB (AAC69525) of S. pneumoniae (25), CpsB (AAK29648) of S. agalactiae (36), and PhpA (WP_011554426.1) of Myxococcus xanthus (21), respectively. Additionally, Php1 possesses all the invariant histidine, aspartate, and arginine residues in four conserved motifs. (B) Php1 structural modeling was performed using the Phyre2 algorithm with 97% confidence. The predicted Php1 model (red) was aligned to the YwqE protein from B. subtilis (gray; PDB code 3QY7). Conserved amino acids are indicated. Download FIG S2, EPS file, 2.2 MB. Copyright © 2019 Jung et al. 2019 Jung et al. This content is distributed under the terms of the Creative Commons Attribution 4.0 International license.

Php1 phosphatase activity on *p*-nitrophenyl phosphate (*p*NPP) was inhibited by the tyrosine phosphatase inhibitor Na_3_VO_4_ (Fig. S3). Inhibition of Php1 was also observed with NaF, which, although a classic serine/threonine phosphatase inhibitor, is a feature of Php enzymes (27) (Fig. S3). Inhibition by EDTA (Fig. S3) corroborated the importance of divalent cations for enzyme activity.

10.1128/mBio.02004-19.3 FIG S3 Inhibition of Php1 phosphatase activity. Recombinant Php1 was incubated with 50 mM *p*NPP in a reaction mixture (pH 8.0) containing the inhibitors indicated. Reactions were performed in triplicate, and data are expressed as means ± SE. ****, *P < *0.001. Download FIG S3, EPS file, 0.4 MB. Copyright © 2019 Jung et al. 2019 Jung et al. This content is distributed under the terms of the Creative Commons Attribution 4.0 International license.

### Substrate specificity of Php1.

**FIG 2:**
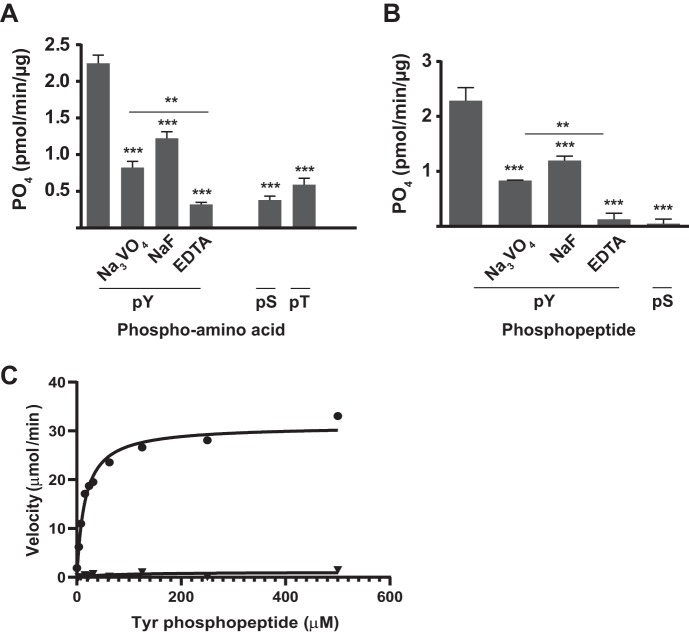
Substrate specificity of Php1. Recombinant Php1 was incubated with 200 μM phospho-amino acids (A) or 100 μM phosphopeptides (B) in the presence or absence of the indicated inhibitors. (C) Saturation kinetics of Php1 with (circles, 4 mM) or without (triangles) Mn^2+^ and tyrosine phosphopeptide substrate at pH 8.0. The graph is a nonlinear fit of the experimental data to the Michaelis-Menten equation. The experiments were performed three times in triplicate, and representative data are shown as means ± SE. **, *P < *0.01; ***, *P < *0.005.

To define the substrate specificity of Php1, phosphatase activity was tested against phospho-amino acids and phosphopeptides using a malachite green phosphate assay. When phospho-amino acids were used as substrates, Php1 was most active against phosphotyrosine, with significantly less phosphate release from phosphoserine and phosphothreonine (Fig. 2A). Php1 activity against phosphotyrosine was significantly inhibited by Na_3_VO_4_, NaF, and EDTA. When phosphopeptides were tested as substrates, Php1 was only active against a tyrosine phosphopeptide, and phosphate release from a serine phosphopeptide was not significantly above background levels (Fig. 2B). Php1 activity against the tyrosine phosphopeptide was also significantly inhibited by Na_3_VO_4_, NaF, and EDTA. The *K_m_* for tyrosine phosphopeptide was 15.56 ± 2.21 μM, with a *V*_max_ of 31.07 ± 1.11 μM/min, a *k*_cat_ of 0.518 ± 0.019 s^−1^, and an enzyme efficiency of 33.29 s^−1 ^μM^−1^ when determined using the Michaelis-Menten equation (Fig. 2C). As a control, Php1 enzyme with no divalent metals was assayed and found to have no activity. These results establish Php1 as an active tyrosine phosphatase *in vitro* with little activity against serine and threonine.

### Dephosphorylation of the Ptk1 tyrosine kinase by Php1.

**FIG 3:**
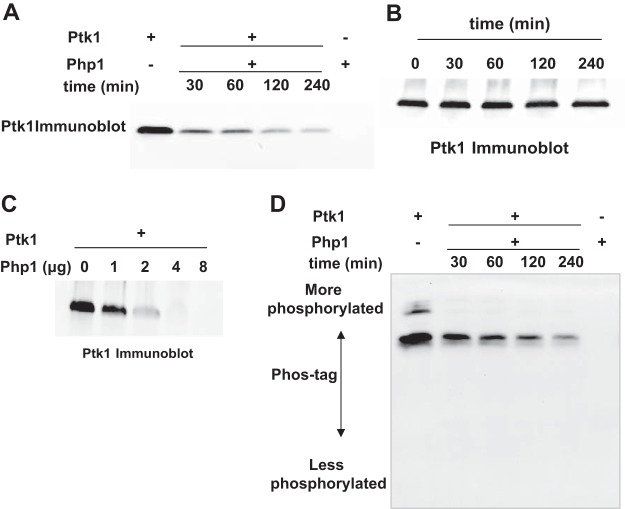
Dephosphorylation of Ptk1 by Php1. Recombinant Ptk1 was incubated with (A) or without (B) recombinant Php1 (5 μg) for the indicated times, and tyrosine phosphorylation of Ptk1 was analyzed by immunoblotting with phosphotyrosine antibodies. (C) Recombinant Ptk1 was incubated with the indicated concentrations of Php1 for 240 min, and tyrosine phosphorylation of Ptk1 was analyzed by immunoblotting. (D) Ptk1 reacted with Php1 as described for panel A and phosphorylation analyzed by Mn^2+^-Phos-tag–SDS-PAGE.

Because of the juxtaposition of the Ptk1- and Php1-encoding genes, we tested the possibility that Ptk1 and Php1 can function as a cognate kinase-phosphatase pair. Recombinant FPtk1 (the C-terminal catalytically active region of Ptk1) purified from Escherichia coli is highly tyrosine phosphorylated, and we have established that residues Y775, Y782, Y784, Y786, Y788, and Y790 in the tyrosine-rich C-terminal cluster are all phosphorylated (13). Tyrosine phosphorylation of Ptk1 decreased after incubation with Php1 in a time- and dose-dependent manner (Fig. 3A and C). Dephosphorylation of Ptk1 required phosphatase enzyme, as the phosphorylation level of Ptk1 was stable in the absence of Php1 (Fig. 3B). Phos-tag electrophoresis showed a rapid transition of Ptk1 from a maximally phosphorylated form to a minimally phosphorylated form in the absence of discrete intermediates (Fig. 3D).

### Mutation of conserved residues of Php1.

**FIG 4:**
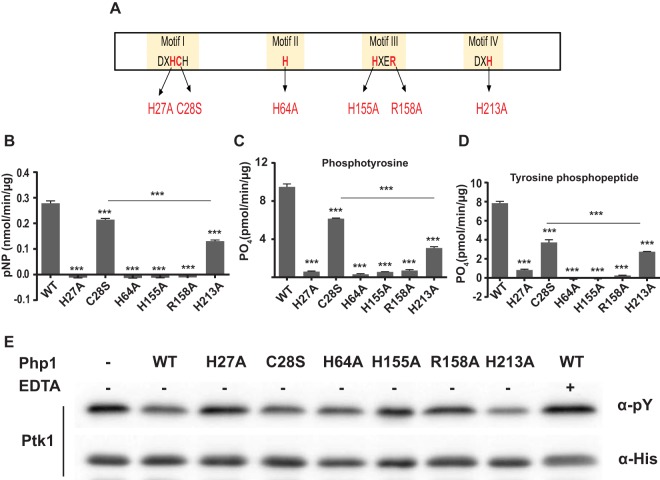
Mutations of conserved His, Cys, and Arg residues in Php1 attenuate phosphatase activity. (A) Schematic representation of Php1 domain structure. Amino acid residues in the four conserved motifs that were mutated are indicated. (B to D) Phosphatase activity of recombinant Php1 wild type (WT) and its mutant proteins was measured using *p*NPP (B), phosphotyrosine (C), or tyrosine phosphopeptide (D) as substrates. The experiments were performed three times in triplicate, and representative data are shown as means ± standard deviations (SD). ***, *P < *0.001. (E) Phosphorylation of Ptk1. Recombinant His-tag Ptk1 was incubated with Php1 WT and its mutant derivatives, and tyrosine phosphorylation of Ptk1 was examined by immunoblotting using antiphosphotyrosine antibody. EDTA was used as a control to inhibit Php1 phosphatase activity. His antibodies were used as a loading control in a duplicate experiment.

Histidine, cysteine, and arginine residues in conserved motifs of the PHP domain of Php1 (depicted in Fig. 4A) were selected for site-directed mutagenesis to determine their roles in Php1 catalytic activity. Among conserved histidine residues, replacement of H27, H64, and H155 with alanine eliminated Php1 phosphatase activity with substrates of *p*NPP, phosphotyrosine, or tyrosine phosphopeptide (Fig. 4B to D), indicating an important role for these residues. An H213A-substituted protein retained 30 to 50% of native enzyme activity with all substrates (Fig. 4B to D). Crystal structure analyses of S. pneumoniae CpsB and B. subtilis YwqE (22, 24) showed that histidine residues in motifs II and IV (corresponding to H64 and H213 in P. gingivalis) are involved in coordination of metal ions. Incomplete inactivation of Php1 by H213A mutation suggests that the role of H213 in metal ion binding is less critical than that of H64. A C28S mutation in Php1 reduced enzyme activity by 30 to 40%. In the structural model (Fig. S2B), C28 faces away from the catalytic site, which may explain why the C28S mutant has less impact on enzyme activity than mutation of the conserved histidine residues. Further, the corresponding substitution in motif I of L. rhamnosus Wzb augmented enzyme activity (23), indicating that the role of the conserved cysteine residue in catalytic activity can vary among different bacteria. In addition to the essential histidine residues, the arginine residue at position 158 in motif III was also required for function, as Php1 with an R158A substitution lost all enzyme activity (Fig. 4B to D). The corresponding arginine residue in S. pneumoniae CpsB also has been reported to be essential for enzyme activity by participating in electron transfer (22, 25).

To investigate the role of these residues in Php1 activity against a P. gingivalis substrate, Ptk1 was incubated with native Php1 or with mutant derivatives. Php1 derivatives C28S, H64, and H213 dephosphorylated Ptk1 to a level similar to that of wild-type Php1 (Fig. 4E), indicating that the phosphatase activity against a cognate kinase is less dependent on these residues than is activity against synthetic substrates. However, mutant derivatives H27A, H155A, and R158A were unable to dephosphorylate Ptk1, supporting an import role for these residues in the Php1-Ptk1 axis.

### Role of Php1 in P. gingivalis single- and dual-species community development and in extracellular polysaccharide production.

**FIG 5:**
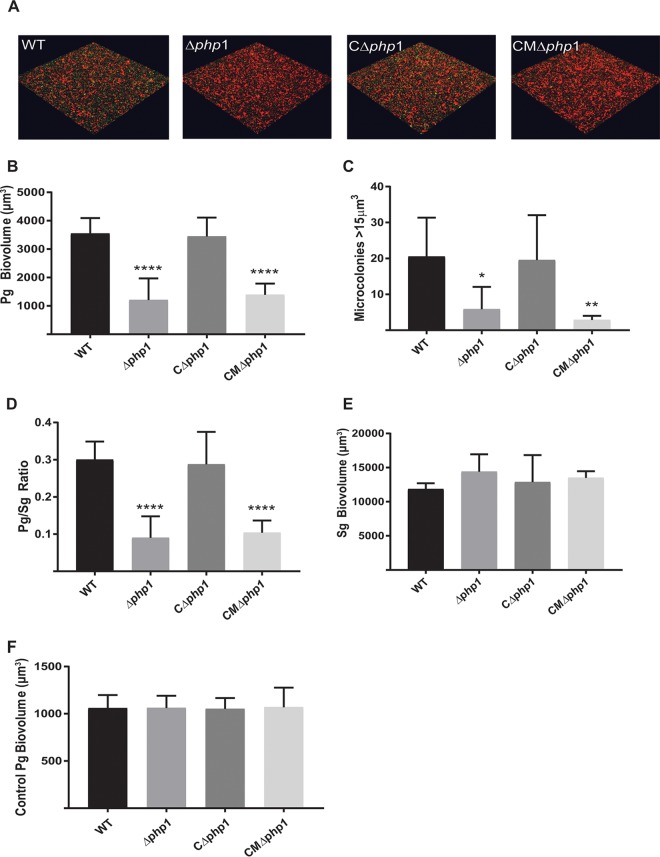
Php1 is required for maximal Porphyromonas gingivalis (Pg)–Streptococcus gordonii (Sg) community development. (A) Confocal scanning laser microscopy visualization of P. gingivalis 33277 (WT), Δ*php1*, Δ*php1 *+* *pTphp1 (CΔphp1), or Δ*php1*+ pTphp1^R158A^ (CMΔ*php1*) (green) strain reacted with a substrate of S. gordonii (red) for 24 h. A series of 0.2-*μ*m-deep optical fluorescent *x-y* sections (213 by 213 *μ*m) were collected using a Leica SP8 confocal microscope and digitally reconstructed into a three-dimensional (3D) image. (B) Total P. gingivalis biovolume was obtained with the 3D Find Objects function of Volocity. (C) Numbers of microcolonies using an object size cutoff algorithm of greater than 15 *μ*m^3^ for the total 3D volume for P. gingivalis fluorescence. (D) Ratio of P. gingivalis to S. gordonii biovolume. (E) Total S. gordonii biovolume was obtained with the 3D Find Objects function of Volocity. (F) Biovolume of P. gingivalis in a parallel experiment without the S. gordonii substratum as a control for nonspecific adherence to the glass coverslip. Error bars are SD, *n *=* *3. *, *P < *0.05; **, *P < *0.01; ****, *P < *0.001, each compared to the WT.

**FIG 6:**
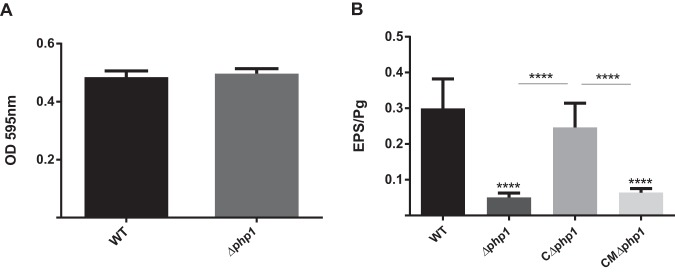
(A) Microtiter plate monospecies biofilm production by P. gingivalis 33277 (WT) and the Δ*php1* mutant at 48 h determined by optical density (OD) of crystal violet staining at 595 nm. (B) Exopolysaccharide produced by P. gingivalis 33277 (WT), Δ*php1*, Δ*php1 *+* *pTphp1 (CΔphp1), or Δ*php1*+ pTphp1^R158A^ (CMΔ*php1*) strain was stained with FITC‐labeled concanavalin A and wheat germ agglutinin. Bacterial cells were stained with Syto 17. Data are ratios of lectin binding (green) to whole-cell staining (red). Error bars are SD, *n* = 3. ****, *P < *0.001.

Ptk1 is required for development of dual-species P. gingivalis-S. gordonii communities in a nutrient-depleted model that allows bacterial signaling-dependent accretion to be distinguished from an increase in biomass due to growth and division (28). Hence, we examined the role of Php1 tyrosine phosphatase activity in community development with S. gordonii. While this assay system did not include saliva or serum, our previous results showed that these biofluids do not have a significant effect on P. gingivalis-S. gordonii community formation (28). As shown in Fig. 5, the Δ*php1* strain demonstrated reduced accumulation on an S. gordonii substrate compared to that of the parental strain. Complementation of the Δ*php1* mutant with the wild-type *php1* allele in *trans* restored the levels of P. gingivalis biovolume in the dual-species accumulations. In contrast, complementation of the Δ*php1* mutant with the R158A mutant *php1* allele, which produces catalytically inactive Php1 protein, failed to restore the ability of P. gingivalis to develop into heterotypic communities with S. gordonii. These results suggest that the Php1 phosphatase activity is required for the development of P. gingivalis microcolonies on a streptococcal substratum. Monospecies biofilm formation by P. gingivalis, however, was unaffected by the loss of Php1 (Fig. 6A). Hence, Php1 is a component of signaling pathways in P. gingivalis that show specificity for mixed-species community development. Ptk1 is also required for maximal extracellular polysaccharide production by P. gingivalis (12), and consistent with this we found that the Php1 mutant strain produced significantly less exopolysaccharide than the parental strain (Fig. 6B). Polysaccharide production was restored in the mutant strain complemented with the wild-type *php1* allele but not in the strain complemented with the R158A mutant *php1* allele. While loss of exopolysaccharide in the encapsulated strain W83 enhances monospecies biofilm production (29), the current data indicate that exopolysaccharide in the nonencapsulated 33277 strain does not play a similar role. Collectively, these results show that a functional Php1-Ptk1 axis is required for both community development with S. gordonii and extracellular polysaccharide production.

**FIG 7:**
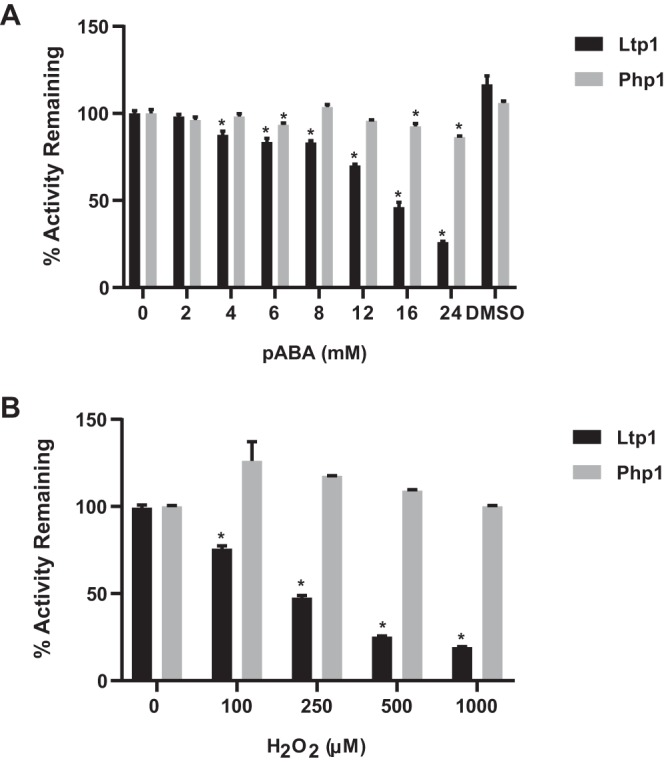
Inhibition of Php1 activity by streptococcal metabolites. Recombinant Php1 was reacted with pABA (A) or H_2_O_2_ (B) at the concentrations indicated, and activity against *p*NPP was measured. Data are expressed relative to activity in the absence of inhibitor. Dimethyl sulfoxide (DMSO) is the vehicle control for pABA. Data are means from three biological replicates ± SE. *, *P < *0.001.

In contrast to Php1, the Ltp1 phosphatase of P. gingivalis restricts community development with S. gordonii (14). The question then arises as to how the two phosphatases are differentially utilized in P. gingivalis in the context of a dual-species community. Previous studies found that para-amino benzoic acid (pABA), secreted by S. gordonii, downregulates the expression of *ltp1* but not *php1*, consequently stimulating community development (11). Additionally, LMW-PTPs such as Ltp1 can also act as redox sensors and are highly sensitive to inhibition by H_2_O_2_ (30), and analogues of amino-benzoic acid are competitive inhibitors of tyrosine phosphatases (31, 32). S. gordonii enzymes Cbe and SpxB, which synthesize pABA and H_2_O_2_, respectively, are known to influence community development with P. gingivalis (11, 28). Hence, we tested the effect of pABA and H_2_O_2_ on the activity of Ltp1 and Php1. Both H_2_O_2_ and pABA inhibited Ltp1 but not Php1 (Fig. 7), indicating that the action of S. gordonii-secreted compounds will favor the development of communities with P. gingivalis through selective inhibition of Ltp1.

### Phosphorylation of Php1 by Ptk1.

**FIG 8:**
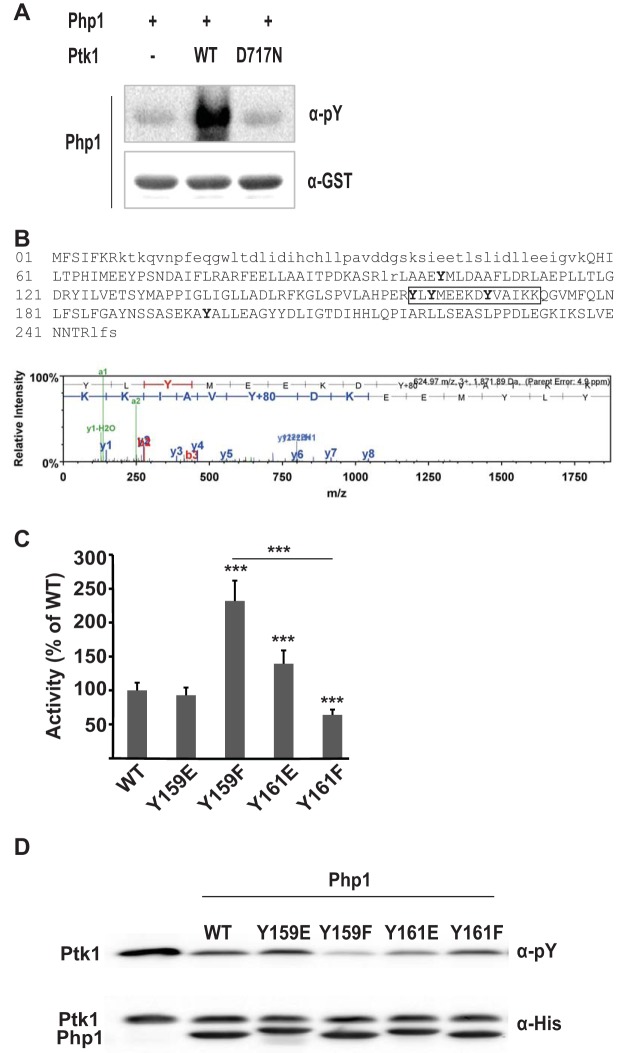
Phosphorylation of Php1 by Ptk1 modulates activity. (A) Recombinant Php1 was incubated with recombinant Ptk1 wild type (WT) or Ptk1 with a mutation in its Walker B domain (D717N) in the presence of 5 mM ATP for 1 h. Tyrosine phosphorylation of Php1 was analyzed by immunoblotting using phosphotyrosine antibodies and GST-tag antibodies as a loading control. (B, upper) Mass spectroscopy sequence coverage for Php1. High-confidence MS/MS data for 20 unique peptides comprising 61 exclusive unique and 119 total spectra were used to assign sequence coverage for 185 (uppercase) of 247 amino acids, achieving 75% coverage. Nondetected residues are in lowercase. Phosphorylated residues are shown in boldface. (Lower) Example of MS/MS spectra used to assign site-specific phosphorylation to MS/MS. (C) Phosphatase activity of recombinant Php1 wild-type (WT) and mutant derivatives was measured against *p*NPP, and the activity of Php1 mutants is shown relative to that of the WT. Data are means ± SD from three biological replicates. ***, *P < *0.001. (D) Recombinant Ptk1 was incubated with/without Php1 WT and mutant derivatives, and tyrosine phosphorylation of Ptk1 was examined by immunoblotting using phosphotyrosine antibodies and His-tag antibodies as a loading control.

We have reported previously that Ptk1 can phosphorylate Ltp1 (13); thus, we tested whether Php1 also acts as a substrate of Ptk1. Tyrosine phosphorylation of Php1 increased after incubation with FPtk1 in the presence of ATP, whereas catalytically inactive FPk1 with a D717N mutation in the Walker B domain failed to phosphorylate Php1 (Fig. 8A). To identify the tyrosine phosphorylation sites, Php1 phosphorylated by Ptk1 was analyzed by mass spectrometry, which identified five tyrosine residues as targets of Ptk1 (Fig. 8B). As phosphorylated Y159 and Y161 residues are close to R158 in motif III, which is required for enzyme activity, we examined whether phosphorylation at these sites affects the phosphatase activity of Php1. Y159 and Y161 were replaced with glutamate or phenylalanine to mimic a phosphorylated or unphosphorylated status, respectively. Php1 Y159F showed 2-fold higher phosphatase activity with *p*NPP substrate than the wild-type Php1, whereas Php1 Y159E had activity similar to that of the wild type (Fig. 8C). In contrast, the Y161F mutation slightly decreased the Php1 activity while Php1 Y161E exhibited increased activity. Similar results were obtained with Ptk1 as a substrate (Fig. 8D). Phosphorylation/dephosphorylation of Php1 at Y159 and Y161 may, therefore, represent a means to fine-tune activity and suggests a reciprocal regulatory relationship with Ptk1.

### Role of Php1 in P. gingivalis *in vivo* pathogenicity.

**FIG 9:**
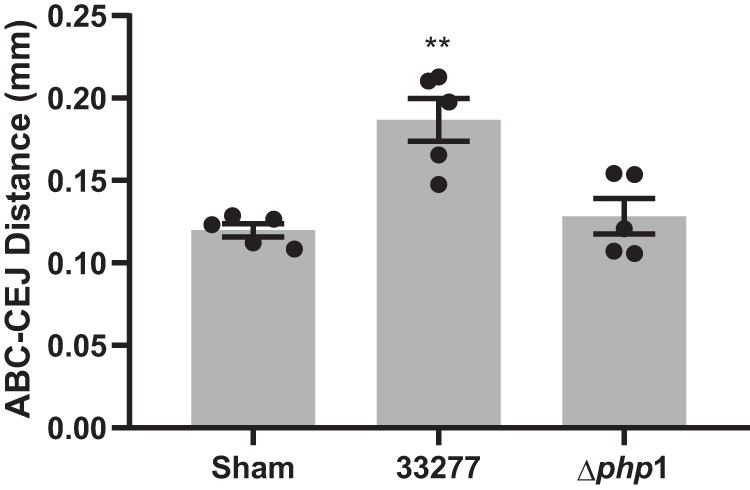
Php1 is required for virulence *in vivo*. Alveolar bone loss in mice following infection with parental (33277) and Δ*php1* mutant P. gingivalis strains was determined by μCT analysis. Reconstructed images of the maxillary molars were analyzed along the sagittal slice to determine the distance between the ABC (alveolar bone crest) and the CEJ (cementoenamel junction) relative to the root length of the tooth. The data are the averages and scatter for each mouse from 12 measurements across both the first and second molars relative to the sham-treated mice and are expressed as means ± SE. **, *P < *0.01 by one-way analysis of variance relative to sham infection.

The contribution of Php1 to the virulence of P. gingivalis was evaluated by measuring alveolar bone loss in a murine model. Mice infected with the parental P. gingivalis developed alveolar bone loss not seen in the sham-treated animals (Fig. 9). Deletion of *php1* significantly reduced the ability of P. gingivalis to induce bone loss, demonstrating Php1 is essential for optimal virulence in a murine model of infection.

## DISCUSSION

Posttranslational modifications of proteins rapidly and reversibly impact conformation, localization, activity, and interaction with other macromolecules, thereby providing a flexible interface connecting intracellular pathways with extracellular stimuli (33). Regulation of tyrosine phosphorylation in bacteria is mediated mainly by bacterial protein-tyrosine (BY) kinases in conjunction with three families of protein-tyrosine phosphatases: the low-molecular-weight phosphatases (LMW-PTPs), a family of small acidic enzymes also found in eukaryotes; the eukaryotic-like phosphatases (PTPs), which are dual-specific phosphatases that also display activity against phosphoserine and phosphothreonine; and the DNA polymerase and histidinol phosphate phosphoesterase (PHP) family (15, 34). In addition to tyrosine phosphatases, PHP proteins also include other types of enzymes, such as histidinol phosphatases, DNA polymerases, and families of uncharacterized proteins in bacteria and eukaryotes (35). Bacterial protein tyrosine phosphatase-encoding genes are often arranged in operons next to BY kinase-encoding genes, which constitute their substrates (15, 36, 37). In the operon of Gram-negative bacteria, a gene immediately upstream of a BY kinase gene usually encodes an LMW-PTP (15). On the other hand, in Gram-positive bacteria, a PHP family tyrosine phosphatase is encoded by a gene immediately upstream or downstream of a BY kinase gene, and LMW-PTPs are often encoded by distantly located genes (19, 36, 38). While Myxococcus xanthus contains a PHP enzyme, P. gingivalis thus far is the first Gram-negative organism possessing both functional LMW and PHP tyrosine phosphatases and with the same operon arrangement as that found in Gram-positive bacteria.

Crystal structures of CpsB, an S. pneumoniae PHP-PTP, and YwqE, a B. subtilis PHP-PTP, have revealed distorted triosephosphate isomerase (TIM) barrel structures composed of several α-helices and parallel β-strands with three metal ions in their active sites (22, 24). Consistent with other PHP family enzymes (21, 25, 26), the Php1 tyrosine phosphatase in P. gingivalis displayed metal ion dependence, primarily for manganese. Attenuation of Php1 activity by mutations of the conserved histidine residues also supports the essentiality of divalent ions, as the corresponding residues in CpsB and YwqE are involved in the coordination of metal ions in their active sites (22, 24). These results suggest that conditions affecting the concentration of divalent ions in saliva and gingival crevicular fluid influence Php1 activity in P. gingivalis. Additionally, Php1 was optimally active at alkaline pH, and the alkaline pH (7.5 to 8.0) of gingival crevicular fluid (39) may therefore provide a favorable environment for Php1 activity. Php1 activity of P. gingivalis in inflamed gingival tissues may be further enhanced as gingival inflammation raises the pH of subgingival pockets up to pH 8.7 (39).

Php1 was capable of dephosphorylation of the chromosomally adjacent tyrosine kinase Ptk1. Hence, P. gingivalis has a unique configuration whereby two tyrosine phosphatases (Php1 and Ltp1) can act on a BY kinase (Ptk1). BY kinases such as Ptk1 possess a cluster of tyrosine residues in the C-terminal domain which are autophosphorylated (13, 40). Phosphorylation in this region is required for kinase function (40), and it is thought that no single tyrosine residue is essential for activity (41). However, it is unclear whether the overall level of tyrosine phosphorylation or a specific combination of tyrosine residue phosphorylation controls kinase activity. The results of our Phos-tag electrophoresis analysis indicate that tyrosine phosphatases switch Ptk1 between high and low phosphorylated states, more consistent with a model where a threshold level of phosphorylation is required for activity.

Although both Php1 and Ltp1 can dephosphorylate recombinant Ptk1 *in vitro*, the (de)phosphorylation axes controlled by the two phosphatases are functionally distinct. Ltp1 participates in a regulatory pathway that constrains P. gingivalis-S. gordonii community development as well as extracellular polysaccharide production (12, 14). In the current study, we found that loss of Php1 diminished the ability of P. gingivalis to accumulate with S. gordonii. In addition, although not organized into a discrete capsule in strain 33277, active Php1 enzyme was required for maximal exopolysaccharide production. The activity of Ltp1, but not Php1, was inhibited by pABA and H_2_O_2_, suggesting that as Php1 is found mainly in Gram-positive organisms, it has evolved resistance to common Gram-positive metabolites. Hence, metabolic cues produced by S. gordonii and sensed by P. gingivalis serve to stimulate exopolysaccharide production and the development of dual-species communities, within which stress responses are reduced, indicative of a mutualistic interaction (42, 43). In dental biofilms, P. gingivalis is frequently in close association with oral streptococci, which may have provided the evolutionary driver for a PHP family phosphatase in order to maintain tyrosine phosphorylation/dephosphorylation ratios in the presence of streptococcal metabolites. Differential properties of the two phosphatases may result from dephosphorylation of different P. gingivalis substrates involved in community development and exopolysaccharide production.

Php1 can be phosphorylated by Ptk1 on two tyrosine residues, 159 and 161. While tyrosine phosphorylation in the DPY or DPYY motif of LMW-PTPs has been reported in E. coli, Mycobacterium tuberculosis, Staphylococcus aureus, and eukaryotic cells (44–47), to our knowledge, this is the first report of phosphorylation of a PHP family phosphatase. The phosphatase activity of Php1 was differentially affected by phosphorylation. Increased activity of Php1 Y159F and decreased activity of Php1 Y161F suggest that the phosphorylation of tyrosine 159 is inhibitory, and the phosphorylation of tyrosine 161 might stimulate activity. Tyrosine 159 is conserved in PHP-PTPs of *Streptococcus* species or altered to asparagine in PHP-PTPs of other Gram-positive bacteria (see Fig. S2 in the supplemental material), whereas tyrosine 161 is not a conserved residue among PHP-PTPs, indicating a potential P. gingivalis-specific regulatory role.

P. gingivalis is a well-recognized pathogen in periodontal diseases (48, 49); however, virulence is expressed in the context of heterotypic communities (5, 6). In the murine alveolar bone loss model, the endogenous mouse microbiota can provide the community context for P. gingivalis-induced disease (50). P. gingivalis exopolysaccharide can also be involved in heterotypic community development and is necessary for virulence of the organism in murine models of disease (51–53). Here, we found that loss of Php1 rendered P. gingivalis unable to induce bone loss in a murine model. These results substantiate the importance of Php1 for the pathophysiology of the organism, effectuated, at least in part, through control of community development and exopolysaccharide production by the Php1-Ptk1 signaling axis. Additionally, in a recent study using a random transposon insertion library, we found that insertional inactivation of either *php1* or *ptk1* significantly reduced fitness in an epithelial colonization model (54), indicating that the P. gingivalis Php1-Ptk1 axis also plays an important role in the interaction with host epithelial barriers and may represent a master regulator of properties important in colonization and virulence.

## MATERIALS AND METHODS

### Bacterial culture.

Porphyromonas gingivalis ATCC 33277 (33277) was cultured anaerobically at 37°C in Trypticase soy broth (TSB) supplemented with 1 mg/ml yeast extract, 5 μg/ml hemin, and 1 μg/ml menadione. When appropriate, tetracycline (1 μg/ml) and/or erythromycin (10 μg/ml) were added to the medium. Streptococcus gordonii DL-1 was cultured anaerobically at 37°C in brain heart infusion broth supplemented with 5 mg/ml yeast extract. Escherichia coli strains were grown aerobically at 37°C with shaking in Luria-Bertani broth containing 100 μg/ml ampicillin or 50 μg/ml kanamycin when required.

### Construction of mutant and complemented strains.

Allelic exchange Δ*php1* mutants were generated as previously described (55). Briefly, upstream and downstream fragments of *php1* (PGN_1525) were amplified by PCR using the primers listed in Table S1 in the supplemental material, and the fragments were fused to *ermF* using the PCR fusion technique. The resulting constructs were electroporated into electrocompetent P. gingivalis, and transformants were selected on TSB plates supplemented with erythromycin. The expression of the upstream and downstream genes PGN_1524 and PGN_1526 in the Δ*php1* strain was comparable to that in the parent strain when analyzed with reverse transcription-PCR (RT-PCR) (data not shown), and there was no difference in growth rate between parent and mutant strains in TSB (data not shown).

10.1128/mBio.02004-19.4 TABLE S1 Primers used in this study. Download Table S1, DOCX file, 0.02 MB. Copyright © 2019 Jung et al. 2019 Jung et al. This content is distributed under the terms of the Creative Commons Attribution 4.0 International license.

To generate the CΔ*php1* complemented strain, the promoter region upstream of PGN_1523 (12) and DNA sequence containing the coding region of *php1* were amplified by PCR and fused together using the PCR fusion technique. The construct was confirmed by sequencing and cloned into pT-COW (56), and the resulting plasmid, pTphp1, was transferred into the Δ*php1* strain through conjugation with E. coli S17-1 containing pTphp1. Transconjugants were selected with gentamicin (50 μg/ml), erythromycin, and tetracycline. To generate a Δ*php1* strain that expresses Php1 with an R158A mutation in the motif III of the PHP domain (the CMΔ*php1* strain), a site-specific mutation was introduced into *php1* of pTphp1 using a Q5 site-directed mutagenesis kit (New England Biolabs) and the primers listed in Table S1. The resulting plasmid, pTphp1^R158A^, was confirmed by sequencing and transferred by conjugation into the Δ*php1* strain, and transconjugants were selected with gentamicin, erythromycin, and tetracycline. Expression of *php1* in the wild type (WT) and in the CΔ*php1* and CMΔ*php* mutant strains was confirmed by RT-PCR (Fig. S1).

10.1128/mBio.02004-19.1 FIG S1 Expression of *php1* mRNA in P. gingivalis 33277 (WT), Δ*php1*, CΔ *php1*, and CMΔ *php1* strains. mRNA levels were measured by quantitative RT-PCR and normalized to 16S levels. Expression in the WT was arbitrarily set to 1. Download FIG S1, EPS file, 0.6 MB. Copyright © 2019 Jung et al. 2019 Jung et al. This content is distributed under the terms of the Creative Commons Attribution 4.0 International license.

### Expression and purification of recombinant proteins.

The 33277 *php1* coding region was amplified by PCR using primers listed in Table S1 and cloned into pGEX-4T-1 (GE Healthcare). The resulting plasmid, pGEX-php1, was transformed into E. coli BL21(DE3) Star, and glutathione *S*-transferase (GST)-tagged protein was purified using glutathione resin (GenScript). Recombinant Php1 with a His tag, after cloning of *php1* into pLATE51 (Thermo Fisher), was expressed in E. coli BL21(DE3) Star and purified using nickel-nitrilotriacetic acid agarose (Qiagen). Site-specific mutations were introduced into *php1* of pGEX-php1 or pLATE51-php1 using a Q5 site-directed mutagenesis kit with the primers listed in Table S1. The following mutations in the PHP domain of Php1 were created: H27A (motif I), C28S (motif I), H64A (motif II), H155A (motif III), R158A (motif III), Y159E or Y159F (motif III), Y161E or Y161F (motif III), and H213A (motif IV). All constructs were confirmed by sequencing. Ltp1 and the active domain of Ptk1 (FPtk1; amino acid residues 541 to 821) were produced as described previously (12, 14). Recombinant Php1 and its derivatives were soluble and purified under native conditions. After buffer exchange into Tris-buffered saline (TBS; pH 7.4), the purity of the recombinant proteins was assessed using SDS-PAGE and Coomassie staining.

### Phosphatase activity assay.

To measure phosphatase activity of recombinant Php1, *p*-nitrophenyl phosphate (*p*NPP; New England Biolabs), phospho-amino acids (phosphotyrosine [Sigma], phosphoserine [Sigma], and phosphothreonine [Sigma]), or phosphopeptides (tyrosine phosphopeptide ENDpYINASL [Promega] and serine phosphopeptide DLDVPIPGRFDRRVpSVAAE [Ser/Thr phosphatase substrate I; R&D Systems]) were used as substrates. The phosphatase assays using *p*NPP as a substrate were carried out at 37°C using 2 μg Php1 and 50 mM *p*NPP in a total volume of 50 μl containing 100 mM Tris-HCl (pH 8.0), 150 mM NaCl, 5 mM dithiothreitol (DTT), and 1 mM MnCl_2_ unless otherwise stated. After adding 50 μl of 3 M NaOH to stop the reaction, the release of *p*-nitrophenol (*p*NP) was determined by reading the optical density at 405 nm. When phosphatase activity was measured using phospho-amino acids or phosphopeptides, the reaction was performed at 37°C using 2 μg Php1 and 200 μM phospho-amino acids or 100 μM phosphopeptides in 80 μl of the reaction buffer described above. Phosphate release was determined with a malachite green phosphate assay kit (Sigma) according to the manufacturer’s instructions. Where indicated, sodium orthovanadate (Na_3_VO_4_; Sigma), sodium fluoride (NaF; Sigma), or ethylenediaminetetraacetic acid (EDTA) was added to the reaction buffer. Kinetic parameters were determined from a nonlinear fit of the Michaelis-Menten equation using Prism 6 software (GraphPad), and a standard curve of inorganic phosphate was used.

### Substrate dephosphorylation.

Ptk1 (5 μg) was incubated with 5 μg Php1 in a total volume of 50 μl containing 100 mM Tris-HCl (pH 8.0), 150 mM NaCl, 5 mM DTT, and 1 mM MnCl_2_ at 37°C for 2 h unless otherwise indicated. The reactions were stopped by adding 5× sample buffer and boiling for 10 min. Samples were separated by SDS-PAGE or Mn^2+^-Phos-tag (Wako)–SDS-PAGE and transferred to polyvinylidene difluoride membranes. The membranes were blocked (TBS containing 0.1% Tween 20 [TBST] and 5% bovine serum albumin) at room temperature for 1 h and washed with TBST. Membranes were probed with mouse antiphosphotyrosine antibody (clone PY20; 1:1,000; Sigma), mouse anti-His-tag antibody (Cell Signaling), or rabbit anti-GST-tag antibody (Cell Signaling) at 4°C overnight. After washing (3× TBST), membranes were incubated with a horseradish peroxidase-conjugated anti-mouse IgG or anti-rabbit IgG secondary antibody (1:1,000; Cell Signaling) at room temperature for 1 h. Immunoreactive bands were detected with Pierce ECL Western blotting substrate (Thermo Fisher) and a ChemiDoc XRS^+^ imaging system (Bio-Rad).

### Identification of phosphorylated residues of Php1.

Php1 (5 μg) was incubated with Ptk1 (5 μg) and 5 mM ATP with PhosSTOP proprietary broad-spectrum phosphatase inhibitor cocktail (Sigma) for 30 min. The proteins were separated by SDS-PAGE and stained with Coomassie brilliant blue, and the Php1 protein band was cut from the gel and stored in water with PhosStop. At the University of Michigan Proteomics and Peptide Synthesis core facility, in-gel digestion of Php1 with trypsin was performed using a ProGest robot (Digilab). The gel slice first was washed with 25 mM ammonium bicarbonate followed by acetonitrile. The slice was then reduced with 10 mM DTT at 60°C, followed by alkylation with 50 mM iodoacetamide at room temperature. The protein was then digested with trypsin (Promega) at 37°C for 4 h followed by inactivation using formic acid, and the supernatant was analyzed directly without further processing. The gel digest was analyzed by nano-liquid chromatography-tandem mass spectrometry (LC-MS/MS) with a Waters NanoAcquity high-performance liquid chromatography system interfaced to a ThermoFisher Q Exactive. Peptides were loaded on a trapping column and eluted over a 75-μm analytical column at 350 nl/min; both columns were packed with Luna C_18_ resin (Phenomenex). The mass spectrometer was operated in data-dependent mode, with the Orbitrap operating at 60,000 full width at half maximum (FWHM) and 17,500 FWHM for MS and MS/MS, respectively. The fifteen most abundant ions were selected for MS/MS. Peptide spectra were analyzed using Scaffold 4 (Proteome Software).

### P. gingivalis*-*S. gordonii dual-species community development.

Heterotypic P. gingivalis-S. gordonii communities were generated as previously described (28). Briefly, hexidium iodide (15 μg/ml; Molecular Probes)-labeled S. gordonii (2 × 10^8^ cells) was deposited on a glass coverslip anaerobically at 37°C for 16 h in phosphate-buffered saline (PBS). After removing unattached S. gordonii, 5 (and 6)-carboxyfluorescein succinimidyl ester (CFSE; 4 μg/ml; Molecular Probes)-labeled P. gingivalis parental or mutant strains (5 × 10^7^ cells) were reacted with S. gordonii in prereduced PBS anaerobically at 37°C for 24 h. After washing with PBS, communities were fixed with 4% paraformaldehyde and examined on a confocal microscope (SP8; Leica) using 488-nm and 552-nm lasers for CFSE and hexidium iodide, respectively. Three-dimensional reconstruction and quantification of the volume of P. gingivalis and S. gordonii fluorescence were carried out using Volocity software (Perkin Elmer).

### P. gingivalis monospecies biofilm formation.

P. gingivalis cells (5 × 10^7^) in TSB were incubated at 37°C anaerobically for 48 h in individual wells of 96-well plates. The resulting biofilms were washed, stained with 1% crystal violet, and destained with 95% ethanol, and absorbance at 595 nm was determined.

### Extracellular polysaccharide production.

Exopolysaccharide production was quantified with fluorescent lectins as described previously (14). In brief, P. gingivalis cells were labeled with Syto 17 (Invitrogen) and deposited in individual wells of 96-well plates. Polysaccharide was labeled with fluorescein isothiocyanate (FITC)‐conjugated concanavalin A and wheat germ agglutinin‐FITC (100 μg ml^−1^) for 30 min at room temperature. After washing, fluorescent levels at 488 nm (FITC) and 648 nm (Syto 17) were determined.

### Murine alveolar bone loss.

C57BL/6 mice, 10 to 12 weeks old, were obtained from Jackson Laboratory. Female mice were used, as studies have shown there to be no gender-dependent differences in alveolar bone loss in a periodontal disease model (57). Mice were fed a standard diet with water *ad libitum*. The University of Louisville Institutional Animal Care and Use Committee approved all animal procedures in this study. P. gingivalis 33277 and Δ*php1* strains (10^9^ CFU were suspended in 0.1 ml of sterile PBS with 2% carboxymethylcellulose [CMC]) were orally inoculated into mice at 2-day intervals over a 12-day period. A control group of mice were mock inoculated with PBS and CMC alone. Forty-two days after the last infection, mice were euthanized and skulls were subjected to micro-computed tomography (μCT) scanning (SKYSCAN 1174; Bruker). Bone loss was assessed by measuring the distance between the alveolar bone crest and the cementoenamel junction at 14 interdental points between the first and second maxillary molars.

### Statistics.

Experiments were conducted in triplicate, and the data presented are representative of at least three independent experiments. Prism 6 software was used for statistical analyses. Analysis of variance with Tukey’s *post hoc* test was used to compare more than two groups.
