# Supplementary material for: Porphyromonas gingivalis Tyrosine Phosphatase Php1 Promotes Community Development and Pathogenicity
Source: mBio. 2019 Sep 24;10(5):e02004-19. doi: 10.1128/mBio.02004-19 (PMC6759763; doi:10.1128/mBio.02004-19)
Supplement: TABLE S1 [file mBio.02004-19-st001.docx]

|  | Table S1. Primers used in this study |  |
| --- | --- | --- |
| Name | Primer sequence (5’ – 3’) | Function |
| 1525upF | CCGGTGAGCGAAGCGAAGAA | Generating fragment upstream of *php1* for allelic exchange mutation in *P. gingivalis* ATCC 33277 |
| 1525upR | CCTATTCCTTTAGCTCTTCTTCATGG |  |
| 1525downF | TATCTGACAGATACATTTGTTTGGGTATGAC | Generating fragment downstream of *php1* for allelic exchange mutation in *P. gingivalis* ATCC 33277 |
| 1525downR | CTCGGATCGTGCTATACGTCC |  |
| ermF_1525F | CCATGAAGAAGAGCTAAAGGAATAGGATGACAAAAAAGAA  ATTGCCCG | Generating erythromycin resistance gene for fusion with upstream and downstream fragments of *php1* |
| ermF_1525R | GTCATACCCAAACAAATGTATCTGTCAGATACTACGAAGG  ATGAAATTTTTCAGG |  |
| 1525promoterF | GCATCGGGATCCTTGGGATTGTGATTATCTTTGCG | Generating promoter region of *php1* for cloning into pT-COW |
| 1525promoterR | CGTTTTTCGCTTGAAAATAGAAAACATAATTGATTTATGG  CTTAAAAAAGG |  |
| ptcow1525F | ATGTTTTCTATTTTCAAGCGAAAAACG | For cloning of *php1* into pT-COW |
| ptcow1525R | CGTAGCGTCGACTCCGGCAGTACACGCTGTATTATA |  |
| pgex1525F | CGCGGATCCATGTTTTCTATTTTCAAGCGAAA | Generation of recombinant Php1 with GST-tag |
| pgex1525R | CGCGTCGACGCTAAGAAAACAATCGGGTGTTG |  |
| pLATE1525F | GGTGATGATGATGACAAGATGTTTTCTATTTTCAAGCGAAA | Generation of recombinant Php1 with His-tag |
| pLATE1525R | GGAGATGGGAAGTCATTACTAAGAAAACAATCGGGTGTTG |  |
| php1_H27A_F | GATAGATATAGCGTGTCATCTGTTGCCTGCAGTGG | Generation of recombinant Php1 with a H27A mutation |
| php1_H27A_R | AGGTCAGTCAACCAACCT |  |
| php1_C28S_F | AGATATACACAGCCATCTGTTGCCTG | Generation of recombinant Php1 with a C28S mutation |
| php1_C28S_R | ATCAGGTCAGTCAACCAAC |  |
| php1_H64A_F | ACTCACTCCCGCGATTATGGAGGAATATC | Generation of recombinant Php1 with a H64A mutation |
| php1_H64A_R | ATATGTTGTTTGACACCTATTTC |  |
| php1_H155A_F | CGTATTGGCAGCGCCCGAACGTTATCTATAC | Generation of recombinant Php1 with a H155A mutation |
| php1_H155A_R | GGGGAAAGACCTTTGAATC |  |
| php1_R158A_F | ACACCCCGAAGCGTATCTATACATGGAGGAG | Generation of recombinant Php1 and pT-COW containing PGN_1525 with a R158A mutation |
| php1 _R158A_R | GCCAATACGGGGGAAAGA |  |
| php1_Y159E_F | CCCCGAACGTgagCTATACATGG | Generation of recombinant Php1 with a Y159E mutation |
| php1 _Y159E_R | TGTGCCAATACGGGGGAA |  |
| php1_Y159F_F | CCCCGAACGTttcCTATACATGGAGG | Generation of recombinant Php1 with a Y159F mutation |
| php1 _Y159F_R | TGTGCCAATACGGGGGAA |  |
| php1_Y161E_F | ACGTTATCTAgaaATGGAGGAGAAAGATTATGTC | Generation of recombinant Php1 with a Y161E mutation |
| php1 _Y161E_R | TCGGGGTGTGCCAATACG |  |
| php1_Y161F_F | ACGTTATCTAtttATGGAGGAGAAAGATTATGTCG | Generation of recombinant Php1 with a Y161F mutation |
| php1 _Y161F_R | TCGGGGTGTGCCAATACG |  |
| php1_H213A_F | GACAGATATAGCGCACCTGCAGCCTATAG | Generation of recombinant Php1 with a H213A mutation |
| php1_H213A_R | CCTATGAGATCATAATAGCC |  |
| pLATE1525F | GGTGATGATGATGACAAGATGTTTTCTATTTTCAAGCGAAA | Generation of recombinant Php1 (PGN_1525) with His-tag |
| pLATE1525R | GGAGATGGGAAGTCATTACTAAGAAAACAATCGGGTGTTG |  |
| RT1524 F | TTCTTGGATTCCGTCCCTGC | Reverse-transcriptase PCR primers to determine gene expression |
| RT1524 R | TTGAGCCGGAGTAAGACACG |  |
| RT1525 F | GCGATCAAAAAGCAAGGCGT |  |
| RT1525 R | AGAAGCCTCGGACAGCAATC |  |
| RT1526 F | GGTACGTAGAGAGATGCGCC |  |
| RT1526 R | GGGTATCATAGCAGTCGCCC |  |
| Pg 16S rRNA F | AGGAACTCCGATTGCGAAGG |  |
| Pg 16S rRNA R | TCGTTTACTGCGTGGACTACC |  |
|  |  |  |
